# Structural and functional features of treatment‐resistant depression: A systematic review and exploratory coordinate‐based meta‐analysis of neuroimaging studies

**DOI:** 10.1111/pcn.13530

**Published:** 2023-02-03

**Authors:** Alessandro Miola, Nicola Meda, Giulia Perini, Fabio Sambataro

**Affiliations:** ^1^ Department of Neuroscience University of Padova Padova Italy; ^2^ Padova Neuroscience Center University of Padova Padova Italy; ^3^ Casa di Cura Parco dei Tigli Padova Italy; ^4^ Padova University Hospital Padova Italy

**Keywords:** depression, functional magnetic resonance imaging, magnetic resonance imaging, treatment‐resistant depression, voxel‐based morphometry

## Abstract

**Objectives:**

A third of people suffering from major depressive disorder do not experience a significant improvement in their symptoms even after adequate treatment with two different antidepressant medications. This common condition, termed treatment‐resistant depression (TRD), severely affects the quality of life of millions of people worldwide, causing long‐lasting interpersonal problems and social costs. Given its epidemiological and clinical relevance and the little consensus on whether the neurobiological underpinnings of TRD differ from treatment‐sensitive depression (TSD), we sought to highlight the convergent morphometric and functional neuroimaging correlates of TRD.

**Methods:**

We systematically reviewed the published literature on structural and resting‐state functional neuroimaging of TRD compared to TSD and healthy controls (HC) and performed exploratory coordinate‐based meta‐analyses (CBMA) of significant results separately for each modality and multimodally (“all‐effects”). CBMAs were also performed for each direction and combining both directions of group contrasts.

**Results:**

Out of the initial 1929 studies, only eight involving 555 participants (189 patients with TRD, 156 with TSD, and 210 HC) were included. In all‐effects CBMA, precentral/superior frontal gyrus showed a significant difference between TRD and HC. Functional and structural imaging meta‐analyses did not yield statistically significant results. A marginally significant cluster of altered intrinsic activity was found between TRD and HC in the cerebellum/pons.

**Conclusions:**

Frontal, cerebellar, and brainstem functions can be involved in the pathophysiology of TRD. However, the design and heterogeneity of the (scarce) published literature hinder the generalizability of the findings.

Major depressive disorder (MDD) is one of the leading causes of disability worldwide^1^ and a significant contributor to the global burden of disease, with an estimated 5.0% of the adult population affected (WHO, 2021). Although the pathophysiology of depression remains unclear, MDD is described as a multifactorial and heterogeneous disorder due to social, psychological, and biological factors.^2^, ^3^

However, the Task Force of the World Federation of Societies of Biological Psychiatry (WFSBP) indicates that many different antidepressants are available for the treatment of MDD in adults,^4^ and one‐third of patients with depression do not respond satisfactorily to initial antidepressant treatment. Moreover, 60%–70% fail to achieve complete remission.^5^, ^6^, ^7^

Treatment‐resistant depression (TRD) is defined as a lack of response to at least one antidepressant trial of adequate dose and duration.^8^ Moreover, in the absence of a univocal definition, several descriptions and guidelines have been proposed for defining or categorizing TRD.^9^ Although the failure of two antidepressant trials is currently the most commonly accepted definition of TRD,^10^ the heterogeneous research methodology and previous inconsistent findings limit the ability to empirically test these definitions that continue to be based on consensus rather than being data‐driven.^11^, ^12^, ^13^ TRD is a condition of utmost clinical relevance given the impact of residual depressive symptoms on functioning, the higher risk of recurrence, the lower chances of remission, and the risk of suicide (which is at least twice the rate of those with nonresistant depression), as well as the increased personal and economic burden associated with TRD.^12^, ^14^, ^15^, ^16^ However, the neurobiological bases of TRD are poorly understood. In this context, neuroimaging studies provide a non‐invasive technique to explore structural and functional abnormalities associated with TRD and treatment‐sensitive depression (TSD), leading to a better understanding of the physiopathology and the development of effective treatment strategies.

Neuroimaging studies revealed differences in patients with MDD in brain volume, function, and connectivity of crucial regions involved in emotion processing and mood regulation.^17^, ^18^, ^19^, ^20^ Brain volume changes in patients with MDD involve several cortical and subcortical areas, including the prefrontal regions, the ventrolateral and ventromedial frontal area, the cingulate cortex, the hippocampus, the insula, the cerebellum, and the striatum.^17^, ^20^, ^21^, ^22^, ^23^ In the last decades, resting‐state functional magnetic resonance imaging (rs‐fMRI) has been widely used to study brain function in several psychiatric disorders, including MDD. This technique allows the investigation of brain function during rest based on spontaneous oscillations of neural activity estimated using the blood‐oxygen‐level‐dependent (BOLD) effect. The BOLD signal can provide different measures of brain activity, such as the fractional amplitude of low‐frequency fluctuations (fALFF) or the regional homogeneity (ReHo) of brain clusters. ReHo indexes (such as ReHo based on Kendall's coefficient of concordance—KCC—or ReHo based on Coherence—CoHe) measure the local synchronization (that reflects functional connectivity) of BOLD signals among neighboring voxels. However, differences in sensitivities between techniques have been reported.^24^ More specifically, KCC‐ReHo measures the local temporal synchronization of BOLD signals, and thus it is sensitive to a time lag between the time series (i.e., time series with similar “shape” but out of phase are less intercorrelated with this method). In contrast, CoHe‐ReHo measures the synchronization of time series in a specific frequency domain; thus, it is less sensitive to phase variations (i.e., it is a technique more suitable to investigate phenomena of information transmission characterized by Granger causality). fALFF is an index that reflects the intensity of spontaneous neuronal activity in low frequencies (0.01–0.08 Hz) relative to the entire spectrum. Compared to ALFF, this technique is less sensitive to physiological noise and correlates with the activity of the local field potential. These indexes provide complementary information on spontaneous brain activity and, for this reason, have previously been meta‐analyzed together to study altered intrinsic brain activity in several neuropsychiatric and metabolic disorders.^25^, ^26^, ^27^ Previous functional magnetic resonance imaging (fMRI) reviews and meta‐analyses have highlighted resting‐state functional connectivity changes in the DMN, salience, attention networks, and other circuits involved in cognitive control and emotional processing in MDD.^28^, ^29^, ^30^, ^31^, ^32^ In addition, a recent meta‐analysis indicated that patients with MDD show increased ALFF in the right superior frontal gyrus (SFG) (including the medial orbitofrontal cortex, medial prefrontal cortex [mPFC], anterior cingulate cortex [ACC]), bilateral insula that extends into the striatum and the left supramarginal gyrus. On the other hand, in MDD decreased ALFF has been reported in the bilateral cerebellum, the bilateral precuneus, and the left occipital cortex.^33^

Although several studies have focused on MDD, structural and functional alterations underlying TRD remain under‐reported. In this context, a previous systematic review summarizing the existing literature up to 2016 focused on structural brain changes in patients with TRD compared to MDD and healthy controls (HC). The authors reported some spatial overlap in brain structural changes between milder forms of MDD and TRD. For example, lower gray matter volume (GMV) in the putamen, inferior frontal gyrus, precentral gyrus, angular and postcentral gyri, and specific changes in the parietal white matter tract appeared to be structural abnormalities specific to TRD.^34^ Furthermore, a recent synthesis of 26 neuroimaging studies using different imaging modalities that investigated the neurobiological differences between TRD and TSD in MDD found that alterations of the default mode network (DMN) (reduced functional connectivity within the DMN, and between DMN subcomponents and brain networks, and increased spontaneous neural activity in the DMN) appear to be critical neurobiological features that differentiate treatment response from resistance in MDD.^35^ However, this review did not apply stringent criteria for the selection of the studies, and several of the included investigations were carried out using the same or partially overlapping samples. Furthermore, the high heterogeneity between studies and different outcome measures did not allow the authors to perform a quantitative analysis and made it difficult to draw any general conclusions. The heterogeneity of the brain changes between patients with TRD and TSD does not allow us to distinguish whether a shared continuum (i.e., similar brain changes with greater magnitude) or a different entity (specific pattern of brain abnormalities) model can explain these differences. Finally, the identification of neuroimaging biomarkers for the diagnosis and treatment of TRD could improve the outcome of this clinical population.

In this systematic review, we aimed to summarize the published literature on the structural or functional neuroimaging correlates of TRD with stringent methodological inclusion criteria. Moreover, we conducted an exploratory coordinate‐based meta‐analysis to identify the areas of the brain involved in TRD.

## Methods

### Protocol and search strategy

**Fig. 1:**
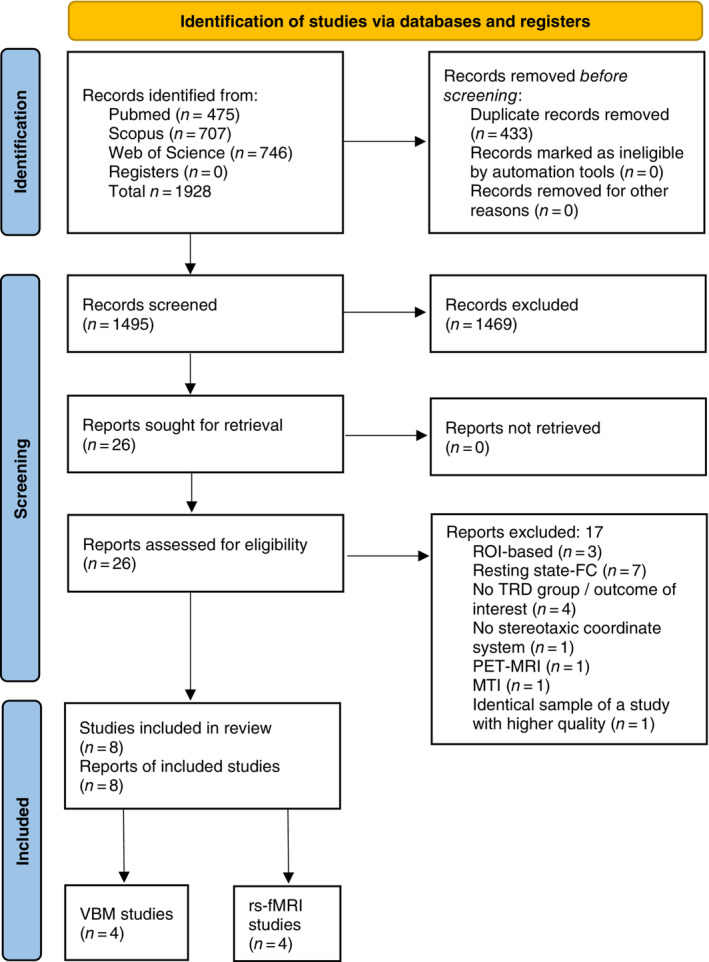
PRISMA flowchart. ROI, Region‐Of‐Interest; FC, Functional Connectivity; TRD, Treatment‐Resistant Depression; PET‐MRI, Positron‐Emission Tomography‐Magnetic Resonance Imaging; MTI, Magnetic Transfer (Ratio) Imaging; VBM, Voxel‐Based Morphometry; rs‐fMRI, resting‐state functional Magnetic Resonance Imaging.

This systematic review followed a pre‐defined protocol available online (https://osf.io/9txa5) and adhered to the procedures of the Preferred Reporting Items for Systematic Reviews and Meta‐Analyses (PRISMA) statement^36^ (see Appendix S1 for details and PRISMA Checklist and Fig. 1). A comprehensive literature search was performed in PubMed, Scopus, and Web of Science databases with the following keywords: “(“TRD“ or “treatment‐resistant depression” or “refractory depression“) and (“MRI” or “imaging” or “magnetic resonance”)”. Moreover, the reference lists of included papers were screened by snowball search and relevant reviews on the subject were consulted as a possible source of eligible studies.

### Eligibility

Case–control, experimental, cross‐sectional and prospective studies were considered eligible. Studies were included if (1) major depressive episodes were diagnosed according to the criteria listed in the Diagnostic and Statistical Manual (DSM) or the International Classification of Diseases (ICD); (2) the authors defined treatment‐resistance as the failure to respond to at least two antidepressant trials^37^, ^38^; (3) compared the structural or functional indexes of the resting state of patients with treatment‐resistant depression (primary diagnosis) to those of patients with treatment‐sensitive depression (TRD *vs*. TSD) or to healthy controls (TRD *vs*. HC); (4) the authors reported the spatial coordinates of the structural or functional contrasts between groups and their stereotaxic space; (5) the findings were corrected, or enough data were available to ensure a correction for multiple comparisons; (6) image acquisition covered the entire brain (including the cerebellum); (7) the samples did not show evidence of serious medical or neurological comorbid conditions; (8) articles were written in English. Studies that compared *a priori* defined regions of interest (ROI) volumes or functional measures or that did not apply a correction to mitigate type I errors at the whole‐brain level were excluded. Studies investigating seed‐based functional connectivity were also excluded due to heterogeneity in seed localization and analytical pipelines. Lastly, task‐based fMRI investigations were not included, as the aim of the current review was to summarize the available literature on the *intrinsic* alterations that can be attributed to treatment‐resistant depression. Commentaries, editorials, and reviews were also excluded. In general, we adhered to the following guidelines.^39^, ^40^

All articles published until the 23rd of April 2022 were included, while no publication status restrictions were imposed.

### Data extraction

Each reference was independently screened by at least two researchers (A.M. and N.M.), and any disagreement was discussed between the two. Whenever it was not possible to make a decision, a third researcher was involved in the discussion (F.S.). The data from the full‐text articles were retrieved and entered into a spreadsheet. Study design, country of study, the definition of resistance to treatment, sample size, demographics, rating scales, the age of onset, duration of the illness, medical or neurological comorbidities, treatment, magnetic resonance imaging scanner, spatial coordinates, stereotaxic space, the statistical significance of clusters and follow‐up time were extracted.

### Study quality

The quality assessment was conducted independently by two researchers (A.M., N.M.) with the Imaging Methodology Quality Assessment Checklist (adapted from^41^) on the following parameters: subjects, imaging acquisition and analysis, and results and conclusions (Supplementary Materials, Table S4). Any persisting disagreements on the quality of the included studies were resolved by a senior researcher (F.S.).

### Exploratory Data Analysis

We performed an exploratory coordinate‐based meta‐analysis (CBMA) using the activation likelihood estimation (ALE) approach implemented in GingerALE (version 3.0.2).^42^ Briefly, we extracted the peak coordinates (foci) of the clusters of significant differences for the contrasts of interest for each study in Talairach space. If the coordinates were reported in MNI space, they were converted to Talairach space using the Lancaster transformation (icbm2tal) available in the software. The foci were then modeled as 3D Gaussian probability distributions centered on the given coordinates to account for spatial uncertainty using a Gaussian kernel width proportional to the study sample size.^43^ For each study, a modeled activation (MA) map was created from the convolved foci using the more dilated mask. All MAs were combined in a single ALE score image that reflects the spatial convergence of the foci.^44^ The significance of the ALE scores was then tested against a null distribution of randomly distributed activation using 1000 permutations. Maps were thresholded using a cluster‐level inference of *p* < 0.05 family‐wise error (FWE) corrected for multiple comparisons and a cluster‐forming threshold of *p* < 0.001. To further investigate clusters that were significant but survived multiple comparison correction, we also performed an exploratory CBMA only with a voxel‐wise threshold of *p* < 0.0001.^44^ First, we performed CBMAs for both voxel‐based morphometry and resting‐state index experiments involving both medicated and nonmedicated patients at the time of the MRI scan that compared the group with treatment‐resistant depression (TRD) with the group with treatment‐sensitive depression (TSD), or the TRD group with healthy controls (HC), independently. Then, the coordinates of all included studies were pooled to conduct an all‐effects exploratory meta‐analysis.^45^ To identify a neurobiological signature of TRD, where neuroimaging changes colocalize (“all‐effects” meta‐analysis),^45^ we combined functional and morphometric neural changes between TRD and a reference group entered into a multimodal CBMA. Indeed, the all‐effects ALE CBMA analysis was previously used to concatenate foci from different experimental contrasts performed on the same subject group.^45^ The rationale of this analysis relies on the fact that morphometric alterations in a brain cluster (e.g., increase in volume) could be or not be accompanied by compensatory functional changes (e.g., increased volumes might represent compensation to a reduced cluster activity and/or the other way around). Thus, given that most of the studies reviewed herein adopted a unimodal approach (i.e., investigated either volumetric changes or functional index differences between groups) and, therefore, could yield only a limited sensitivity for the neural alterations in TRD, we opted to conduct an additional all‐effects CBMA to identify any neural substrate (irrespective of imaging modality) implicated in this condition. For this reason, in an all‐effects meta‐analysis, the studies were not stratified according to the direction of contrast (i.e., hyper or hypoactivation or increase/decrease in GMV can be specified). Specifically, two multimodal CBMAs were performed: one on TRD *vs*. TSD and the other on the comparison of TRD *vs*. HC, respectively. Eventually, we conducted four post‐hoc CBMAs: TRD > TSD, TSD > TRD, TRD > HC, and HC > TRD.

## Results

A total of 1929 studies were identified from Scopus, Pubmed, and Web of Science. After duplicate removal, 1495 abstracts were selected, of which 26 full‐text articles were retrieved for in‐depth assessment. The list of the 18 studies excluded after full‐text assessment, and the reasons for exclusion, are reported in the Supplementary Material Appendix S1. A total of eight studies, four VBM and four rs‐fMRI, were included in the systematic review and exploratory CBMAs (the characteristics of the included studies are reported in Table 1). Overall, this review gathered 555 participants (244 participants in fMRI and 311 in VBM studies, respectively): 189 patients with TRD (79 fMRI/110 VBM), 156 with TSD (77 fMRI/79 VBM), and 210 HC (88 fMRI/122 VBM). The eight studies reported at least one focus of significant difference between the groups. Six studies (75%) were carried out in South‐East Asia (two in Japan, one in Taiwan, and four in China; three studies were from the same group of authors), one in Spain, and one in France.

**Table 1:** Characteristics of the studies included in the exploratory coordinate‐based meta‐analysis

Study	Country	N (TRD/TSD/HC)	Age (TRD)	Female (TRD)	Diagnostic criteria	TRD pharmacological criteria	Age of onset, years, mean ± SD (TRD/TSD)	Duration of illness, months, mean ± SD (TRD/TSD)	Rating scales	Median score, mean ± SD (TRD/TSD)	Treatment %, TRD (TSD)	Quality score
Sandu *et al*., 2017^51^	France	41/40/44	47.1 ± 8	63.4%	DSM‐IV	2 AD from different classes for 1 month	28.26 ± 9.92/28.06 ± 10.90	18.90 ± 9.21/18.01 ± 11.56 (years)	MADRS	34.20 ± 7.19/12.70 ± 13.78	Antidepressants 46.34 (57.50) Anticovulsivants 29.27 (25.00) Lithium 12.20 (25.00) Antipsychotics 39.02 (22.50) Benzodiazepines 43.90 (37.50)	9
Yamamura *et al*., 2016^57^	Japan	16/16/26	44.6 ± 9.7	37.5%	DSM‐IV‐TR	2 AD	39.3 ± 9.9/42.3 ± 13.1	NA	HRSD17	13.6 ± 3.8/15.4 ± 3.1	TCA 25.00% (−) SSRI 68.75 (100) SNRI 18.75 (−) NaSSA 25.00 (−) Other antidepressants 25.00 (−) Lithium 12.5 (−) Anticonvulsivants 18.75 (−) Antipsychotics 56.25 (−) Stimulants 6.25 (−) Benzodiazepines 87.5 (68.75)	10
Machino *et al*., 2014^56^	Japan	29/0/29	39.6 ± 8.3	44.8%	DSM‐IV	2 AD	34.72 ± 7.56/‐	52.55 ± 57.81/‐	HRSD	13.90 ± 4.33/‐	TCA 51.72 SSRI 41.37 SNRI 27.58 Other antidepressants 34.48 Lithium 20.68 Antipsychotics 31.03 Stimulants 3.44 Anxiolytics 27.58	8.5
Serra‐Blasco *et al*., 2013^55^	Spain	22/22/32	49 ± 8	68.2%	DSM‐IV‐TR	Thase–Rush Index of treatment resistance ≥3	27.4 ± 8.4/29.7 ± 11	NA	HRSD1	21 ± 4.6/16 ± 6.5	SSRI 86 (75) TCA 36 (15) Other antidepressants 57 (0.5) Stabilizers 36 (20) Antipsychotics 45 (10) Benzodiazepines 59 (30)	9
Ma *et al*., 2012*^50^	China	18/17/17	27.4 ± 7.7	38.9%	DSM‐IV	2 AD from different classes for 6 weeks	NA	35.5 ± 49.89/2.59 ± 1.33	HAMD	23.89 ± 3.69/25.58 ± 6.32	*TSDs were treatment naive at the scan*	8.5
Guo *et al*., 2012*^49^	China	23/22/19	27.4 ± 7.7	38.9%	DSM‐IV	2 AD of different classes for 6 weeks	NA	27.43 ± 35.89/ 2.95 ± 1.73	HRSD	24.52 ± 4.17/ 25.89 ± 6.26	*TSDs were treatment naive at the scan*	8.5
Guo *et al*., 2012*^48^	China	18/17/17	27.3 ± 7.2	52.2%	DSM‐IV	2 AD of different classes for 6 weeks	NA	35.5 ± 49.89/2.59 ± 1.33	HRSD	23.89 ± 3.69/25.58 ± 6.32	TCA 27.77 SSRI 33.33 SNRI 22.22 Anticonvulsivants 11.11 Lithium 5.55 Antipsychotics 11.11 *TSDs were treatment naïve at scan*	8.5
Wu *et al*., 2011^52^	China	22/22/26	35 ± 13	31.8%	DSM‐IV	2 AD of different classes for 6 weeks	NA	103 ± 65/32 ± 64	HRSD	22.0 ± 3.5/23.2 ± 4.8	NA	9

Abbreviations: *, it marks the studies from the same group of authors; AD, AntiDepressants; Age and Female data (TRD) are referred to the group of patients with TRD; Cohe‐ReHo, Coherence‐based Regional Homogeneity; fALFF, fractional Amplitude of Low‐Frequency Fluctuations; HAMD, Hamilton Depression Rating Scale; HC, Healthy controls; HRSD, Hamilton Rating Scale for Depression; MADRS, Montgomery‐Asberg Depression Rating Scale; N, sample size (TRD + TSD + HC); NA, Not available; NaSSA, Noradrenergic and specific serotonergic antidepressants; rs‐fMRI, resting‐state functional Magnetic Resonance Imaging; SNRI, Serotonin‐norepinephrine reuptake inhibitors; SSRI, Selective serotonin reuptake inhibitors; TCA, Tricyclic antidepressants; TRD, Treatment‐Resistant Depression; TSD, Treatment‐response depression; VBM, Voxel‐Based Morphometry.

### Quality assessment

The quality of the included studies was measured according to an adapted version of the Imaging Methodology Quality Assessment Checklist (Checklist in Supplementary Material Appendix S1—see also^46^, ^47^) is reported in Table 1. The Risk‐of‐bias assessment is summarized in Figure S1 (see Supplementary Material Appendix S1).

### Treatment‐resistance definition in the included studies

In our pooled sample of studies, treatment resistance was defined as non‐responsiveness (less than 50% reduction in the Hamilton Depression Rating Scale score) to at least two six‐week or longer trials of antidepressant medication by 5 out of 8 studies.^48^, ^49^, ^50^, ^51^, ^52^ However, Sandu *et al*., 2017^51^ used a trimmed version of the minimum duration of treatment, 1 month, referring to the study by Berlim & Turecki (2007).^53^ The three following studies also specified the level of treatment‐resistance according to Thase & Rush, 1997:^54^ Serra‐Blasco *et al*. (2013) referred to TRD stage ≥3,^55^ Machino *et al*. (2014) specified the percentage of patients in stage 2 or 3,^56^ and Yamamura *et al*. (2016) recruited patients with TRD of at least stage 2.^57^ Notably, a study conducted by Sandu *et al*. (2017)^51^ included unipolar and bipolar TRD in the treatment‐resistance group.

### Voxel‐based morphometry

Regarding volumetric differences between patients with treatment‐resistant unipolar or bipolar depression compared to patients with treatment‐sensitive depression, a previous report identified that patients with a treatment‐resistant condition had higher GMV in the left and right amygdala than patients with TSD, regardless of the underlying mood disorder.^51^ Machino *et al*., 2014 found smaller volumes of ventral and dorsal ACC, frontal gyrus, cerebellar crus, and vermis in patients with TRD than in HC. Moreover, the right superior temporal gyrus volume was associated with the severity of the rumination.^56^ Compared to HC, patients with TRD displayed a broad set of clusters of reduced GMV in the anterior cingulate gyrus, superior, medial and inferior frontal gyri, the insula, the parahippocampal gyrus, and the transverse temporal gyrus.^55^ Lastly, Ma and colleagues revealed that, compared to HC and TSD, patients with TRD exhibited reduced GMV in the caudate.^50^ We performed an exploratory CBMA (irrespective of the contrast direction, for example, TRD > TSD or TSD > TRD) on VBM data (patients with TRD/TSD/HC = 110/79/90) none of the foci (*n* = 19) reported by the studies (*n* = 4) (Table 2) had a significant spatial overlap.^50^, ^51^, ^55^, ^56^ No significant clusters of morphometric differences could be identified between TRD and TSD or HC. Even when the FWE correction was not applied, no clusters could be identified.

**Table 2:** Imaging acquisition characteristics and cluster coordinates identified by the studies included in the exploratory coordinate‐based meta‐analysis

Study	Imaging Power Field	Type of analysis	Contrast	Peak‐voxel localization (MNI)		
Sandu *et al*., 2017^51^	1.5T	VBM		*x*	*y*	*z*
			TRD > TSD	28	4	−20
			TRD > TSD	40	−1	−15
			TRD > TSD	−34	2	−18
Yamamura *et al*., 2016^57^	3 T	rs‐fMRI, fALFF		*x*	*y*	*z*
			TRD > TSD	54	30	0
			TRD > TSD	−30	−90	24
			TRD > TSD	21	−18	12
			TRD > TSD	54	−45	27
			TRD > TSD	0	−45	27
			TRD > HC	57	30	3
			TRD > HC	48	−78	27
			TRD > HC	3	−51	6
			TRD > HC	0	−57	48
			TRD > HC	21	−21	3
			TRD > HC	−9	−42	−33
			HC > TRD	−42	−18	63
			HC > TRD	15	−102	−3
			HC > TRD	−9	−99	−6
			HC > TRD	−15	−27	63
			HC > TRD	−30	−24	42
Machino *et al*., 2014^56^	1.5 T	VBM		*x*	*y*	*z*
			HC > TRD	−3	9	22
			HC > TRD	20	−85	−24
Serra‐Blasco *et al*., 2013^55^	3 T	VBM		*x*	*y*	*z*
			HC > TRD	5	34	49
			HC > TRD	5	51	36
			HC > TRD	2	60	17
			HC > TRD	−14	7	36
			HC > TRD	−6	−3	34
			HC > TRD	−11	−5	65
			HC > TRD	−48	12	−0
			HC > TRD	−56	9	13
			HC > TRD	−9	38	46
			HC > TRD	−24	−10	−32
			HC > TRD	−59	−20	14
			HC > TRD	−61	−29	18
Ma *et al*., 2012*^50^	1.5 T	VBM		*x*	*y*	*z*
			TSD > TRD	7	6	10
			HC > TRD	61	−34	−3
Guo *et al*., 2012*^49^	1.5 T	rs‐fMRI, ReHo		*x*	*y*	*z*
			TSD > TRD	−39	−66	−42
			TRD > TSD	−30	−93	0
			HC > TRD	−18	−12	72
			HC > TRD	−9	−39	−39
Guo *et al*., 2012*^48^	1.5 T	rs‐fMRI, fALFF		*x*	*y*	*z*
			TRD > TSD	−9	−78	−21
			TRD > TSD	−3	12	−12
			TSD > TRD	−18	−69	0
			TRD > HC	−42	−42	−36
			TRD > HC	6	33	−9
			HC > TRD	−6	−39	−21
			HC > TRD	−51	−69	6
Wu *et al*., 2011^52^	3 T	rs‐fMRI, Cohe‐ReHo		*x*	*y*	*z*
			TRD > TSD	57	−39	0
			TRD > TSD	36	−21	12
			TRD > TSD	−3	−15	45
			TSD > TRD	−12	−78	54
			TSD > TRD	−48	0	27
			TRD > HC	33	−12	18
			TRD > HC	−9	48	12
			TRD > HC	−12	−12	66
			TRD > HC	15	48	0
			TRD > HC	57	−39	3
			HC > TRD	−54	9	21
			HC > TRD	−33	−78	−18
			HC > TRD	−45	−24	51
			HC > TRD	54	−42	48
			HC > TRD	−24	−66	54

Abbreviations: Cohe‐ReHo, Coherence‐based Regional Homogeneity; fALFF, fractional Amplitude of Low‐Frequency Fluctuations; HC, Healthy controls; MNI, Montreal Neurological Institute (brain template); rs‐fMRI, resting‐state functional Magnetic Resonance Imaging; TRD, Treatment‐Resistant Depression; TSD, Treatment‐responsive depression; VBM, Voxel‐Based Morphometry.

### Resting‐state fMRI

Two of the four resting‐state fMRI studies included in this systematic review used fALFF to measure the BOLD signal intensity. Guo *et al*. (2012) identified widespread differences in ALFF values among patients with TRD, TSD, and HC. Specifically, patients with TRD showed higher values in the posterior lobes of the cerebellum and the DMN (ACC and medial frontal gyrus) than in HC. In comparison, lower ALFF values were found in the visual recognition circuit.^48^ Yamamura and co‐workers showed that TRD had increased values of the right thalamic fALFF relative to TSD. In contrast, the fALFF signal in the right inferior frontal gyrus (IFG), inferior parietal lobule (IPL), and cerebellar vermis was higher in patients with TRD than in TSD / healthy control.^57^ The remaining two studies used either ReHo or Cohe‐ReHo. Wu *et al*. (2011) showed that patients with TRD have more widely distributed ReHo cerebral alterations compared to patients with TSD. Also, TRD exhibited higher ReHo in the right middle temporal gyrus, the right insula, and the middle cingulate than TSD. Clusters with lower ReHo were found only in the left hemisphere and included the precuneus and inferior frontal gyrus, lateral inferior frontal gyrus, prefrontal gyrus, precuneus, and intraparietal and superior parietal lobule. Furthermore, the ReHo values of the left precuneus were significantly inversely correlated with disease duration.^52^ On the other hand, Guo *et al*. (2012) identified reduced Cohe‐ReHo in the bilateral superior frontal gyrus and cerebellum in TRD compared to HC. Compared with TSD, TRD showed a higher Cohe‐ReHo in the left fusiform gyrus.^49^

The overall CBMAs on resting‐state functional data (number of foci = 27, number of experiments = 4), yielded a single cluster at a cluster‐forming threshold of *p* < 0.0001 for the comparison between TRD and HC (TRD/HC = 79/88), without an FWE correction. This cluster (from the studies by Yamamura *et al*.^57^ and Guo *et al*.^49^) belongs to the cerebellum with a peak of the signal at coordinates [*x*; *y*; *z*; −10; −36; −32], a volume of 232 mm^3^, an ALE value of 0.013, and *p* = 0.0000041. Post‐hoc contrast direction‐specific CBMAs (i.e., evaluating whether this cluster pinpointed a hyper‐ or hypoactivation of the cerebellum in TRD) did not show any significant cluster (Table 2 for more details).

### All‐effects (Functional imaging and Morphometry) coordinate‐based meta‐analysis

**Fig. 2:**
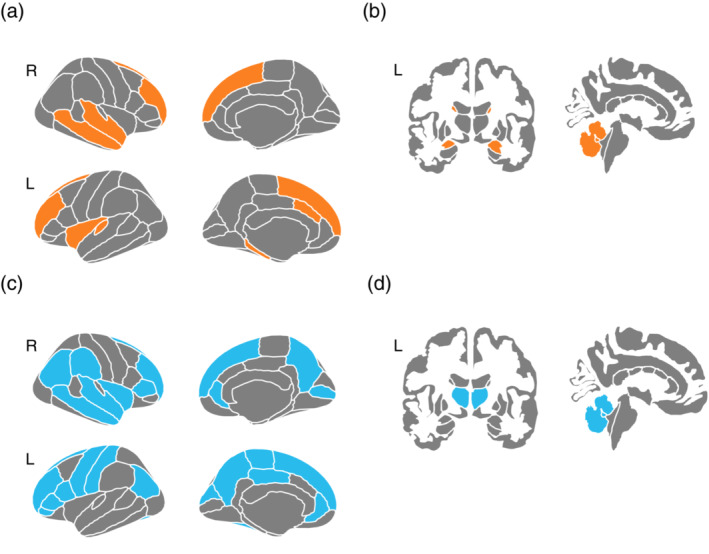
Brain regions altered in Treatment‐Resistant Depression (TRD) in each neuroimaging modality. The results of morphometric (a, b) and resting functional magnetic resonance (c, d) studies are displayed on the Desikan–Killiany atlas. On the leftmost side, the lateral and medial cortical surfaces are displayed for each hemisphere (a, c); on the rightmost side, the coronal and sagittal projections are shown (b, d). Significantly altered morphometry (gray matter volume) in cortical (a; caudal anterior cingulate cortex, rostral middle frontal gyrus, superior frontal gyrus, insula, parahippocampal gyrus, transverse, superior and middle temporal gyrus) and subcortical (b; amygdalae, caudate nuclei, and cerebellum) regions in TRD relative to treatment sensitive depression (TSD) and healthy controls (HC) in the included studies, respectively (here reported in orange). Significantly altered resting‐state activity (low‐frequency oscillations, regional homogeneity) in the cortical (c; right superior and middle temporal gyrus, right inferior frontal gyrus pars triangularis, right middle and inferior occipital gyrus, right supramarginal gyrus, right insula, left middle cingulate, left inferior frontal gyrus, left cuneus, left precentral and postcentral gyrus, left paracentral lobule, left fusiform gyrus, bilateral inferior parietal lobule, bilateral precuneus, bilateral superior frontal gyrus, bilateral anterior cingulate cortex/medial frontal gyrus) and subcortical (d; the thalamic nuclei and cerebellum) regions in TRD relative to HC in the included studies, respectively (reported in blue). The renderings were created using the R‐package *ggseg*.

**Fig. 3:**
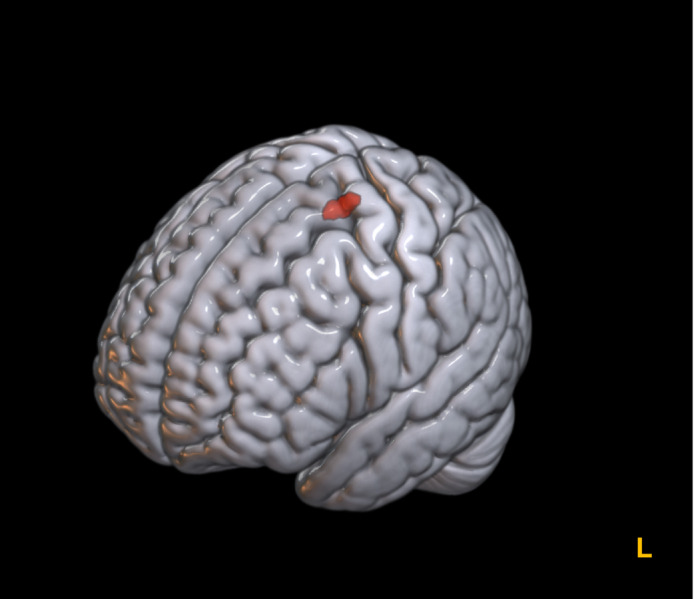
Convergent multimodal neurobiological changes in Treatment‐Resistant Depression (TRD). L, left. A significant convergence of neural differences estimated using a multimodal approach, including brain morphometry (gray matter volume) and resting‐state functional magnetic resonance imaging (low‐frequency fluctuations) in patients with TRD relative to healthy controls (HC) was found in a cluster in the left precentral/superior frontal gyrus (at *x*, *y*, *z* = [−12; −16; 62], here reported in red). The results are FWE corrected at 0.05 at the cluster level with cluster‐forming probability at *p* < 0.001 The probability map thresholded is rendered on a standard brain in Montreal Neurological Institute (MNI). The rendering was created using MricroGl.

Lastly, we pooled all resting‐state fMRI and VBM studies (Fig. 2, Figure S2 available in Supplementary Material Appendix S1) and analyzed all group contrasts together and separately (e.g., TRD *vs*. TSD; TRD *vs*. HC, etc.). All‐effects exploratory CBMA in TRD relative to HC included seven experiments with 314 participants (TRD/HC = 148/166) and comprised 42 brain foci.^48^, ^49^, ^50^, ^52^, ^55^, ^56^, ^57^ This analysis yielded a significant cluster in the superior frontal gyrus/precentral gyrus (peak signal at coordinates [−12; −16; 62], volume = 576 mm^3^, ALE = 0.0135, *p* = 0.00001, p cluster‐level FWE *p* < 0.05; Fig. 3), and resulted from the overlap of three foci from three different studies.^49^, ^52^, ^55^ For an all‐effects CBMA, the studies are not usually stratified according to the direction of contrast (so no hyper‐ or hypoactivation or increase/decrease in volume can be specified, see Materials and Methods section for further details). No other contrasts (i.e., TRD *vs*. TSD) produced significant results.

### Patient comorbidities in the included studies

Serious medical or neurological comorbidities were exclusion criteria for this study. Specifically, 7 out of 8 reports excluded patients with serious physical or mental disorders other than MDD. One study excluded patients only for physical comorbidities and recruited patients with unipolar or bipolar depression.^51^ Indeed, Sandu *et al*. (2017) also enrolled patients with BD in TRD and non‐TRD samples^51^ (for more details, see Supplementary Material, Section S1).

### Patient medication status in the included studies

The status of the medication, which is one important factor in modulating brain structure and function (see below) varied greatly among the studies. For a detailed description of the antidepressants (and other psychiatric medications) used in each study, see Table 1. Two studies^48^, ^49^ explicitly stated that patients with TSD did not have a history of antidepressant treatment (i.e., the MRI scan was performed before starting any antidepressant medication). The study by Ma and colleagues (2012) included drug‐naïve patients in their first major depressive episode.^50^ On the other hand, Sandu *et al*. (2017) defined TSD as a medication‐induced remission of MDD, including patients who were mostly treated at the time of the scan (74/81) for unipolar and bipolar depression in both TRD and non‐TRD samples—nonetheless, the authors found no difference between unipolar and bipolar TRD.^51^ In the study conducted by Wu and colleagues,^52^ previous exposure to antidepressants or mood episodes in the TSD group was unclear. However, the authors explicitly stated that the MRI scan was performed before the start of an antidepressant trial. Yamamura *et al*. (2016) recruited patients with TSD, who were untreated or treated with a single antidepressant with an insufficient dose and duration.^57^ The two remaining investigations^55^, ^56^ did not detail previous treatment with antidepressant medications prior to the scan.

## Discussion

To our knowledge, this is the first systematic review to apply stringent criteria to summarize the findings of CBMA‐eligible studies on structural or functional resting neuroimaging correlates of treatment‐resistant depression (TRD). This study included eight MRI studies: four used a voxel‐based morphometry (VBM) approach, and four conducted functional MRI at rest. A total of 555 participants were included: 189 patients with TRD, 156 with TSD, and 210 HC. Three main findings emerged. First, we found no significant clusters of VBM difference between TRD and TSD or HC. Second, a single cluster in the cerebellum/pons showed a marginal difference between TRD and HC when rs‐fMRI studies were combined. Lastly, a significant cluster was identified in the precentral/superior frontal gyrus when comparing patients with TRD with HC in multimodal analyses.

Despite the growing interest and the number of MRI studies published on TRD in the last decade, our systematic review yielded mainly divergent findings, probably due to the heterogeneity underlying the TRD condition itself and to different methodological approaches.

Regarding the definitions of TRD, all the studies included people who lacked clinical improvement after the use of at least two different antidepressants prescribed at adequate doses for 4 weeks to 6 weeks. However, possible reasons for these scant findings are related to the heterogeneity of the selected study populations in the different studies, which refers to depression being described as a heterogeneous disorder.^58^, ^59^ The condition itself can be diagnosed by at least 256 unique symptom combinations, which leads to 1030 individual symptom profiles that meet the criteria for MDD.^58^, ^60^ Various clinical presentations combined with low interrater reliability make the diagnosis of depression heterogeneous *per se*.^61^ Moreover, the populations of the included studies differed in symptom severity (i.e., mild, moderate, and severe), diagnostic categories including bipolar *vs*. MDD, and illness history (i.e., recurrent *vs*. single episode). Furthermore, there are different specifiers for the course or presentation of symptoms, configuring several distinct depression subtypes based on clinical symptoms.^62^

Although all of the structural neuroimaging studies included in this review and exploratory CBMA identified several anatomical abnormalities between TRD, TSD, and HC, most of these results failed to be replicated consistently. In line with a previous systematic review that suggested a lack of convergence on the structural brain changes underlying TRD *vs*. TSD and HC,^34^ here we confirm that there are no significant clusters of morphometric differences between these samples. Indeed, compared to HC, both TRD and TSD showed a decreased GMV in the right middle temporal gyrus (MTG), while only TRD exhibited reduced GMV in the bilateral caudate. Compared to TSD, GMV was reduced in the bilateral caudate^50^ and increased in the bilateral amygdala in TRD. This latter increase did not differ between unipolar and bipolar depression and was not related to medication.^51^ Furthermore, compared to HC, TRD showed smaller GMV in the left dorsal and right ventral ACC, the right superior frontal gyrus, the right cerebellum, and the cerebellar vermis, and a positive correlation between rumination and GMV in the right superior temporal gyrus.^56^ Despite several differences identified in these studies, none had significant overlap between studies; therefore, no brain morphometric difference could be considered a characteristic of TRD.

The rs‐fMRI studies converged on a single cluster in cerebellum/pons showing a functional alteration between TRD and HC that was driven by two studies with opposite direction changes. However, this cluster did not survive the correction for multiple comparisons. Moreover, individual studies that were meta‐analyzed reported divergent directions of their contrast results. In general, TRD showed widespread alterations of ReHo relative to HC and TSD. Compared to TSD, ReHo was higher in the right middle temporal gyrus, the right insula, and the middle cingulate, and lower in the left precuneus and the left inferior frontal gyrus in TRD, respectively. ReHo was higher in small clusters in the medial prefrontal and parahippocampal areas and lower in the left PFG in TSD compared to HC, respectively.^52^ Furthermore, TRD showed higher values of fALFF in the right inferior frontal gyrus, the inferior parietal lobule and the cerebellum vermis, compared to TSD and HC.^57^ Widespread differences in ALFF values emerged between TRD, TSD, and HC throughout the cerebellum, the visual recognition circuit (middle temporal gyrus, middle/inferior occipital gyrus, and fusiform), the striatum circuit (putamen), the DMN circuit (ACC and medial frontal gyrus) and the risk/action circuit (inferior frontal gyrus). In particular, TRD and TSD showed ALFF differences mainly in the cerebellum, the visual network, and the DMN.^48^ In another study by the same authors, TRD exhibited a lower Cohe‐ReHo in the bilateral superior frontal gyrus and the left cerebellum compared to HC. In contrast, in TSD, lower Cohe‐ReHo was observed in the bilateral superior frontal gyrus. Compared to TSD, Cohe‐ReHo was lower in the bilateral cerebellum and higher in the left fusiform gyrus in TRD.^49^ Thus, the superior frontal gyrus showed lower resting‐state brain activity in TRD and TSD,^49^ suggesting its role as a putative trait marker for MDD.

Notably, we found that intrinsic neural activity showed a marginal difference between TRD and HC in a cluster located in the cerebellum/brainstem. Although the cerebellum has traditionally been considered a region of motor control and coordination,^63^, ^64^, ^65^ it is also involved in cognitive and emotional processes, as well as mood regulation.^65^, ^66^, ^67^ In line with our findings, the cerebellar vermis has also been described as the “limbic cerebellum” due to its connections with limbic structures and its relevance for mood disorders.^68^, ^69^, ^70^ However, the exact mechanism through which the cerebellum can play a role in the pathophysiology of TRD remains unknown. Patients with cerebellar damage can show the “cerebellar cognitive‐affective syndrome” or the “Schmahmann syndrome”,^71^, ^72^ which includes impaired executive function, visuospatial cognition, emotional affect, and language, suggesting a cerebellar modulation of neural circuits related to the prefrontal, posterior parietal, superior temporal, and limbic cortices.^71^, ^72^ In addition, a previous meta‐analysis revealed that patients with cerebellar lesions were more likely to have depression, emotional blunting, and behavioral difficulties.^73^ Interestingly, the affective syndrome after cerebellar damage is also consistent with several case reports in children and adolescents that show a wide range of mood symptoms, including depression, lack of emotions, and affect dysregulation.^74^, ^75^, ^76^, ^77^ Previous evidence supports a bidirectional anatomical and functional connection of the cerebellum with several brain regions related to mood regulation, including the prefrontal cortex, hypothalamus, reticular system, hippocampus, amygdala, and septal nuclei.^70^, ^78^, ^79^, ^80^, ^81^ Moreover, reciprocal connections linking the cerebellum with brain stem nuclei containing neurotransmitters involved in mood regulation, including serotonin, norepinephrine, and dopamine, have been described.^82^ These neuroanatomical substrates can explain the role of the cerebellum in influencing the constitutive aspects of affect, including autonomic function, arousal, expression, and cognitive processing of emotions.^83^ Consistently, compared with TSD, TRD had lower Cohe‐ReHo values in the bilateral cerebellum, and abnormal neural activity in the cerebellum has been proposed as a marker to differentiate TRD from TSD with high sensitivity and specificity (83% and 86%, respectively).^49^

The multimodal (“all‐effects”) meta‐analyses identified neural abnormalities in the precentral gyrus/superior frontal gyrus in TRD relative to HC. These analyses combined structural and functional imaging studies regardless of the contrast direction, thus capitalizing on the different sensitivity of the techniques and highlighting any neurobiological change that may be associated with TRD. Notably, all the fMRI studies included in this analysis included abnormal activations in the frontal lobe in TRD relative to TSD or HC, including the medial and inferior frontal gyrus,^49^, ^52^ the superior frontal gyrus,^48^ and in proximity to the precentral gyrus.^57^ However, this focus did not contribute to our results. A morphometric study identified several foci belonging to the frontal lobe, left medial, inferior, and right superior, in TRD, one of which contributed to the meta‐analytical cluster.^55^ Previous literature has shown that the activity of frontal regions is altered in depressive disorders,^84^ which can underlie altered emotion regulation and attentional processes.^85^, ^86^ Moreover, volumetric reduction in the frontal regions has also been found in depression,^22^, ^87^, ^88^ and, more specifically, in TRD.^34^, ^35^ Altered frontal morphometry is believed to be associated with abnormal (hyper)activation of the same region in a compensatory process after gray matter loss or to reflect treatment refractoriness.^57^ Additionally, dysfunction of the frontal regions has also been described in the co‐occurrence of depression and anxiety disorders.^89^ Indeed, generalized anxiety disorder, posttraumatic stress disorder, and MDD are highly co‐morbid.^90^, ^91^ However, identifying such disorder‐specific correlates is particularly challenging because neuroimaging investigations rarely exclude (or purposefully match for) these comorbidities. In line with our findings, a recent meta‐analysis investigating specific neural correlates for such highly comorbid disorders found disorder‐specific GMV reductions in fronto‐limbic and cerebellar regions in MDD, fronto‐temporal areas in anxiety disorders, and fronto‐occipital regions in posttraumatic stress disorder,^23^ respectively. Given the high degree of comorbidity between anxiety and depression, as well as the role of anxiety in predicting a poorer response to treatment with antidepressant medications for depressive disorders,^92^ frontal regions could represent a target for future antidepressant treatments. Indeed, previous evidence suggests that functional neuroimaging can identify the dynamics of the brain network involved in response to antidepressant treatment.^93^ For example, in a proof of mechanism study, acute citalopram administration modulated static and dynamic resting‐state connectivity of the mPFC in HC.^94^ Furthermore, when administered to patients with MDD, citalopram normalized the connectivity of the precuneus and amygdala with the DMN, restoring the functional activity pattern reported in healthy controls.^95^ Moreover, the antidepressant response has been associated with increased connectivity between frontal and limbic brain regions, possibly resulting in greater inhibitory control over the emotional processing circuits.^93^ Specifically, frontal regions, ACC, dorsolateral prefrontal cortex, orbitofrontal cortex, amygdala, and hippocampus have been reported as potential target regions for the prediction of antidepressant responses in MDD,^96^ and normalization of aberrant activity in the amygdala and ventral ACC represents one of the most consistently replicated biomarkers of antidepressant response.^97^ Interestingly, task‐based and non‐triggered fMRI investigations can converge in case of non‐response at least in some brain areas such as the amygdala, where increased neural activity tends to normalize after clinical response, and the opposite is true in case of nonresponse.^97^ Lastly, fMRI studies have yielded promising results not only for measuring the response to treatment in MDD but also for predicting remission after SSRI treatment. Remarkably, a resting‐state fMRI study found that in first‐episode medication‐free patients with MDD, signal changes in the caudate, occipital, and temporal cortices measured 5 h after the first dose of escitalopram were able to predict clinical remission after 8 weeks of treatment with this drug.^98^

This study has several limitations that should be considered when interpreting the results. First, the cross‐sectional design and relatively small sample size of the included neuroimaging studies limit our ability to make inferences about the causality and generalization of our findings. Indeed, recruiting treatment‐naïve patients for studies on treatment‐resistant mood disorders can be challenging.^99^ Second, MDD commonly occurs with other physical conditions, including obesity and type 2 diabetes^100^, ^101^, ^102^, ^103^, ^104^ and mental disorders, most notably anxiety disorders and substance use disorders^105^, ^106^, ^107^ that were not systematically investigated in previous neuroimaging studies and can hinder the response to treatment, leading to pseudo‐resistance. Third, patients differed in variables associated with the longitudinal course of their illness, including the age of onset, frequency and duration of depressive episodes, stage of illness, and cognitive impairment, which are not systematically reported and can represent notable sources of heterogeneity of our findings. Fourth, another important factor underlying the heterogeneity of the results could include the treatment status, which varied significantly between the included studies, contributing to the difficult characterization of patients with TSD *vs*. those with TRD. Indeed, the role of morphometric changes following antidepressant treatment has been previously demonstrated in patients with MDD.^108^, ^109^, ^110^ For example, a selective increase in GMV in the hippocampus has been reported after acute treatment and remission in patients with nonmedicated MDD prospectively treated with citalopram.^110^ Fifth, from a methodological perspective, imaging‐related settings differed between the studies included in this work, such as magnetic field strength (which ranged from 1.5T to 3T), slice thickness, and analysis pipelines. Regarding study‐related differences, inclusion criteria for HC, the clinical course of the disorder (e.g., acute *vs*. chronic), and statistical methods could potentially affect our conclusions. Despite the scarcity of statistically significant results, this work underscores the need for replicable high‐quality TRD neuroimaging research studies. Future studies should (i) carefully match patients with TRD with patients with TSD for similar physical comorbidities and other psychiatric co‐diagnoses, possibly with the exclusion of patients with a history of substance use disorders; (ii) use rigorous research approaches with the pre‐registration of the research protocol, including preprocessing and analysis pipelines and accurate power analyses; (iii) apply conservative statistical approaches (e.g., corrections for multiple comparisons, whole‐brain *vs*. ROI level). Future prospective longitudinal neuroimaging investigations are needed in patients with MDD who are naïve to treatment to differentiate biomarkers that may be predictors of resistance to treatment from those associated with disease progression.

In conclusion, this systematic review and exploratory meta‐analysis supports the role of frontal regions in the pathophysiology of TRD. Although the high clinical heterogeneity of TRD and the different methodological approaches used to study the functional neuroimaging of this condition, we also highlighted that the cerebellum/pons can be a candidate brain region for the identification of treatment‐resistant depression. Further studies using multimodal and task‐based approaches are warranted to better characterize the role of this region in treatment resistance to depression.

## Author contributions

Alessandro Miola, Nicola Meda, and Fabio Sambataro conducted the literature review. Fabio Sambataro and Giulia Perini guided the areas for discussion. Alessandro Miola, Nicola Meda, and Fabio Sambataro conducted the statistical analysis. Alessandro Miola and Nicola Meda wrote the first draft of the manuscript. All authors have contributed equally to the critical revision of the manuscript.

## Funding information

The authors received no specific funding for this work.

## Conflict of interest

The authors declare no conflict of interest.

## Supporting information


**Appendix S1.** Supplementary Information

## Data Availability

All data can be retrieved from the manuscript or supplementary material.
