# Supplementary material for: Structural and functional features of treatment‐resistant depression: A systematic review and exploratory coordinate‐based meta‐analysis of neuroimaging studies
Source: Psychiatry Clin Neurosci. 2023 Feb 3;77(5):252–63. doi: 10.1111/pcn.13530 (PMC11488613; doi:10.1111/pcn.13530)
Supplement: Supplementary file 1 — Appendix S1. Supplementary Information [file PCN-77-252-s001.docx]

Supplementary material

**Structural and functional features of treatment-resistant depression: A systematic review and exploratory coordinate-based meta-analysis of neuroimaging studies**

**Running Title: Neuroimaging of TRD**

Alessandro Miola MD ^1,2,3,^*, Nicola Meda MD ^1,^*, Giulia Perini MD ^1,2,3^, Fabio Sambataro MD PhD ^1,2,4^

1 Department of Neuroscience, University of Padova, Padua, Italy

2 Padova Neuroscience Center, University of Padova, Padua, Italy

3 Casa di Cura Parco dei Tigli, Padova, Italy

4 Padova University Hospital, Padua, Italy

* These authors contributed equally to this work

Corresponding author:

Fabio Sambataro, MD, PhD

Department of Neuroscience (DNS), University of Padova

Via Giustiniani, 3, Padua, Italy

Tel: +390498211980

Email: [fabio.sambataro@unipd.it](mailto:fabio.sambataro@unipd.it)

**Index**

eTable 1. PRISMA checklist.

eTable 2. ABSTRACT checklist.

eTable 3. List of excluded studies after full-text assessment, with reason(s) for exclusion.

eTable 4. Imaging Quality Assessment Checklist – adapted.

eFigure 1. Risk of Bias.

Section 1. Patients’ comorbidities in the included studies.

eFigure 2. Convergent intrinsic neural activity changes in TRD.

eTable 1. PRISMA 2020 checklist.

| **Topic** | **No.** | **Item** | **Location where item is reported** |
| --- | --- | --- | --- |
| **TITLE** |  |  |  |
| **Title** | 1 | Identify the report as a systematic review. | 1 |
| **ABSTRACT** |  |  |  |
| **Abstract** | 2 | See the PRISMA 2020 for Abstracts checklist | 2 |
| **INTRODUCTION** |  |  |  |
| **Rationale** | 3 | Describe the rationale for the review in the context of existing knowledge. | 4, 5 |
| **Objectives** | 4 | Provide an explicit statement of the objective(s) or question(s) the review addresses. | 5 |
| **METHODS** |  |  |  |
| **Eligibility criteria** | 5 | Specify the inclusion and exclusion criteria for the review and how studies were grouped for the syntheses. | 5, 6 |
| **Information sources** | 6 | Specify all databases, registers, websites, organisations, reference lists and other sources searched or consulted to identify studies. Specify the date when each source was last searched or consulted. | 5, 6 |
| **Search strategy** | 7 | Present the full search strategies for all databases, registers and websites, including any filters and limits used. | 5, 6 |
| **Selection process** | 8 | Specify the methods used to decide whether a study met the inclusion criteria of the review, including how many reviewers screened each record and each report retrieved, whether they worked independently, and if applicable, details of automation tools used in the process. | 5-7 |
| **Data collection process** | 9 | Specify the methods used to collect data from reports, including how many reviewers collected data from each report, whether they worked independently, any processes for obtaining or confirming data from study investigators, and if applicable, details of automation tools used in the process. | 6 |
| **Data items** | 10a | List and define all outcomes for which data were sought. Specify whether all results that were compatible with each outcome domain in each study were sought (e.g. for all measures, time points, analyses), and if not, the methods used to decide which results to collect. | 6 |
|  | 10b | List and define all other variables for which data were sought (e.g. participant and intervention characteristics, funding sources). Describe any assumptions made about any missing or unclear information. | 6 |
| **Study risk of bias assessment** | 11 | Specify the methods used to assess risk of bias in the included studies, including details of the tool(s) used, how many reviewers assessed each study and whether they worked independently, and if applicable, details of automation tools used in the process. | 6 |
| **Effect measures** | 12 | Specify for each outcome the effect measure(s) (e.g. risk ratio, mean difference) used in the synthesis or presentation of results. | 6 |
| **Synthesis methods** | 13a | Describe the processes used to decide which studies were eligible for each synthesis (e.g. tabulating the study intervention characteristics and comparing against the planned groups for each synthesis (item 5)). | 5, 6 |
|  | 13b | Describe any methods required to prepare the data for presentation or synthesis, such as handling of missing summary statistics, or data conversions. | NA |
|  | 13c | Describe any methods used to tabulate or visually display results of individual studies and syntheses. | 6, 7 |
|  | 13d | Describe any methods used to synthesize results and provide a rationale for the choice(s). If meta-analysis was performed, describe the model(s), method(s) to identify the presence and extent of statistical heterogeneity, and software package(s) used. | 6, 7 |
|  | 13e | Describe any methods used to explore possible causes of heterogeneity among study results (e.g. subgroup analysis, meta-regression). | NA |
|  | 13f | Describe any sensitivity analyses conducted to assess robustness of the synthesized results. | NA |
| **Reporting bias assessment** | 14 | Describe any methods used to assess risk of bias due to missing results in a synthesis (arising from reporting biases). | NA |
| **Certainty assessment** | 15 | Describe any methods used to assess certainty (or confidence) in the body of evidence for an outcome. | NA |
| **RESULTS** |  |  |  |
| **Study selection** | 16a | Describe the results of the search and selection process, from the number of records identified in the search to the number of studies included in the review, ideally using a flow diagram. | 7, 8; Figure 1 |
|  | 16b | Cite studies that might appear to meet the inclusion criteria, but which were excluded, and explain why they were excluded. | eTable 3 in Suppl. Material |
| **Study characteristics** | 17 | Cite each included study and present its characteristics. | Table 1 |
| **Risk of bias in studies** | 18 | Present assessments of risk of bias for each included study. | eFigure 1 in Suppl. Material |
| **Results of individual studies** | 19 | For all outcomes, present, for each study: (a) summary statistics for each group (where appropriate) and (b) an effect estimate and its precision (e.g. confidence/credible interval), ideally using structured tables or plots. | 8, Table 2 |
| **Results of syntheses** | 20a | For each synthesis, briefly summarise the characteristics and risk of bias among contributing studies. | eFigure 1 |
|  | 20b | Present results of all statistical syntheses conducted. If meta-analysis was done, present for each the summary estimate and its precision (e.g. confidence/credible interval) and measures of statistical heterogeneity. If comparing groups, describe the direction of the effect. | 7, 8, Figure 2, Figure 3 |
|  | 20c | Present results of all investigations of possible causes of heterogeneity among study results. | 7, 8 |
|  | 20d | Present results of all sensitivity analyses conducted to assess the robustness of the synthesized results. | NA |
| **Reporting biases** | 21 | Present assessments of risk of bias due to missing results (arising from reporting biases) for each synthesis assessed. | eFigure 1 in Suppl. Material |
| **Certainty of evidence** | 22 | Present assessments of certainty (or confidence) in the body of evidence for each outcome assessed. | NA |
| **DISCUSSION** |  |  |  |
| **Discussion** | 23a | Provide a general interpretation of the results in the context of other evidence. | 10-13 |
|  | 23b | Discuss any limitations of the evidence included in the review. | 13, 14 |
|  | 23c | Discuss any limitations of the review processes used. | 13, 14 |
|  | 23d | Discuss implications of the results for practice, policy, and future research. | 13-15 |
| **OTHER INFORMATION** |  |  |  |
| **Registration and protocol** | 24a | Provide registration information for the review, including register name and registration number, or state that the review was not registered. | 5 |
|  | 24b | Indicate where the review protocol can be accessed, or state that a protocol was not prepared. | 5 |
|  | 24c | Describe and explain any amendments to information provided at registration or in the protocol. | NA |
| **Support** | 25 | Describe sources of financial or non-financial support for the review, and the role of the funders or sponsors in the review. | 15 |
| **Competing interests** | 26 | Declare any competing interests of review authors. | 15 |
| **Availability of data, code and other materials** | 27 | Report which of the following are publicly available and where they can be found: template data collection forms; data extracted from included studies; data used for all analyses; analytic code; any other materials used in the review. | 15 |

**eTable2.** ABSTRACT checklist.

| **Topic** | **No.** | **Item** | **Reported?** |
| --- | --- | --- | --- |
| **TITLE** |  |  |  |
| **Title** | 1 | Identify the report as a systematic review. | Yes |
| **BACKGROUND** |  |  |  |
| **Objectives** | 2 | Provide an explicit statement of the main objective(s) or question(s) the review addresses. | Yes |
| **METHODS** |  |  |  |
| **Eligibility criteria** | 3 | Specify the inclusion and exclusion criteria for the review. | No |
| **Information sources** | 4 | Specify the information sources (e.g. databases, registers) used to identify studies and the date when each was last searched. | No |
| **Risk of bias** | 5 | Specify the methods used to assess risk of bias in the included studies. | No |
| **Synthesis of results** | 6 | Specify the methods used to present and synthesize results. | No |
| **RESULTS** |  |  |  |
| **Included studies** | 7 | Give the total number of included studies and participants and summarise relevant characteristics of studies. | Yes |
| **Synthesis of results** | 8 | Present results for main outcomes, preferably indicating the number of included studies and participants for each. If meta-analysis was done, report the summary estimate and confidence/credible interval. If comparing groups, indicate the direction of the effect (i.e. which group is favoured). | Yes |
| **DISCUSSION** |  |  |  |
| **Limitations of evidence** | 9 | Provide a brief summary of the limitations of the evidence included in the review (e.g. study risk of bias, inconsistency and imprecision). | Yes |
| **Interpretation** | 10 | Provide a general interpretation of the results and important implications. | Yes |
| **OTHER** |  |  |  |
| **Funding** | 11 | Specify the primary source of funding for the review. | No |
| **Registration** | 12 | Provide the register name and registration number. | No |

*From:* Page MJ, McKenzie JE, Bossuyt PM, Boutron I, Hoffmann TC, Mulrow CD, et al. The PRISMA 2020 statement: an updated guideline for reporting systematic reviews. MetaArXiv. 2020, September 14. DOI: 10.31222/osf.io/v7gm2. For more information, visit: [www.prisma-statement.org](file:///Users/alessandro/Downloads/www.prisma-statement.org)

**eTable 3**. List of excluded studies after full-text assessment, with reason(s) for exclusion.

| DOI | First author, year | Country/region | Reason for exclusion |
| --- | --- | --- | --- |
| 10.2147/NDT.S199456 | (Zhang et al., 2019) | China | ROI-based |
| 10.1016/j.nicl.2019.102064 | (Geugies et al., 2019) | The Netherlands | FC |
| 10.1007/s11682-017-9773-0 | (Hou et al., 2018) | China | FC |
| 10.1148/radiol.2017160820 | (Jia et al., 2017) | China | MTI |
| 10.1016/j.jpsychires.2016.07.011 | (He et al., 2016) | China | FC |
| 10.1016/j.euroneuro.2015.08.009 | (Carceller-Sindreu et al., 2015) | Spain | ROI-based |
| 10.1016/j.euroneuro.2015.04.025 | (Abdallah et al., 2015) | USA | ROI-based |
| 10.3389/fpsyt.2015.00028 | (de Kwaasteniet et al., 2015) | The Netherlands | FC |
| 10.1192/bjp.bp.113.140434 | (Li et al., 2015) | Taiwan | PET-MRI |
| 10.1016/j.pnpbp.2013.01.010 | (Guo et al., 2013b) | China | FC |
| 10.1186/1745-6215-14-224 | (Grieve et al., 2013) | USA/Australia | No pop. of interest |
| 10.1371/journal.pone.0071368 | (Guo et al., 2013a) | China | FC |
| 10.1371/journal.pone.0040968 | (Liu et al., 2012) | China | *Same population of (Ma et al., 2012) |
| 10.1176/appi.ajp.2010.10101419 | (Lui et al., 2011) | China | FC |
| 10.1016/j.neuroimage.2010.11.079 | (Gong et al., 2011) | China | No outcome of interest |
| 10.1016/j.biopsych.2010.07.029 | (Soriano-Mas et al., 2011) | Spain | No pop. of interest |
| 10.1016/j.neuroimage.2009.11.021 | (Li et al., 2010) | Taiwan | No pop. of interest |
| 10.1192/bjp.180.5.434 | (Shah et al., 2002) | Scotland | No template |

**Characteristics of the studies excluded from the coordinate-based meta-analysis and reasons for exclusion.** ROI = Region-Of-Interest; FC = Functional Connectivity; pop = population; PET-MRI = Positron-Emission Tomography-Magnetic Resonance Imaging; MTI = Magnetic Transfer (Ratio) Imaging; * the study uses voxel-based morphometry to identify structural differences between patients with TRD and those with TSD. The sample is identical to the one analysed in Ma et al., 2012 (10.1371/journal.pone.0045263). Nonetheless, the study by Liu F et al., 2011 would not have been included as the slice number, thickness, and inter-slice gap are missing.

**eTable 4.** Imaging Quality Assessment Checklist – adapted

|  | **Category 1: Subjects** | **Score** (0/0.5/1) |
| --- | --- | --- |
| 1 | People with treatment-resistant depression were evaluated, specific diagnostic criteria were applied, and demographic data was reported |  |
| 2 | People with treatment-sensitive depression were evaluated, specific diagnostic criteria were applied, and demographic data was reported. Alternatively, healthy comparison subjects were evaluated, psychiatric and medical illnesses  were excluded and demographic data was reported. |  |
| 3 | Important variables (e.g. age, gender, handedness, height or total brain measures) were checked, either by stratification or statistically |  |
| 4 | Sample size per group > 7 |  |
|  | **Category 2: Methods for image acquisition and analysis** |  |
| 5 | All neuroanatomic measurements were taken without considering group assignment or subject identity |  |
| 6 | Magnet strength > 1T |  |
| 7 | The imaging technique used was clearly described so that it could be reproduced |  |
| 8 | Measurements were clearly described so that they could be reproduced |  |
|  | **Category 3: Results and conclusions** |  |
| 9 | Statistical parameters for significant, and important non-significant, differences were provided |  |
| 10 | Conclusions were consistent with the results obtained and the limitations were discussed |  |
|  | **TOTAL** | /10 |

Adapted Imaging Methodology Quality Assessment Checklist from

Cattarinussi, G., Di Giorgio, A., Wolf, R. C., Balestrieri, M., & Sambataro, F. (2019).

Neural signatures of the risk for bipolar disorder: A meta‐analysis of structural and functional

neuroimaging studies. *Bipolar Disorders*, *21*(3), 215–227. https://doi.org/10.1111/bdi.12720

Original Checklist available from

Shepherd, A. M., Matheson, S. L., Laurens, K. R., Carr, V. J., & Green, M. J. (2012). Systematic

Meta-Analysis of Insula Volume in Schizophrenia. Biological Psychiatry, 72(9), 775–784.

https://doi.org/10.1016/j.biopsych.2012.04.020

**eFigure 1.** Risk of Bias


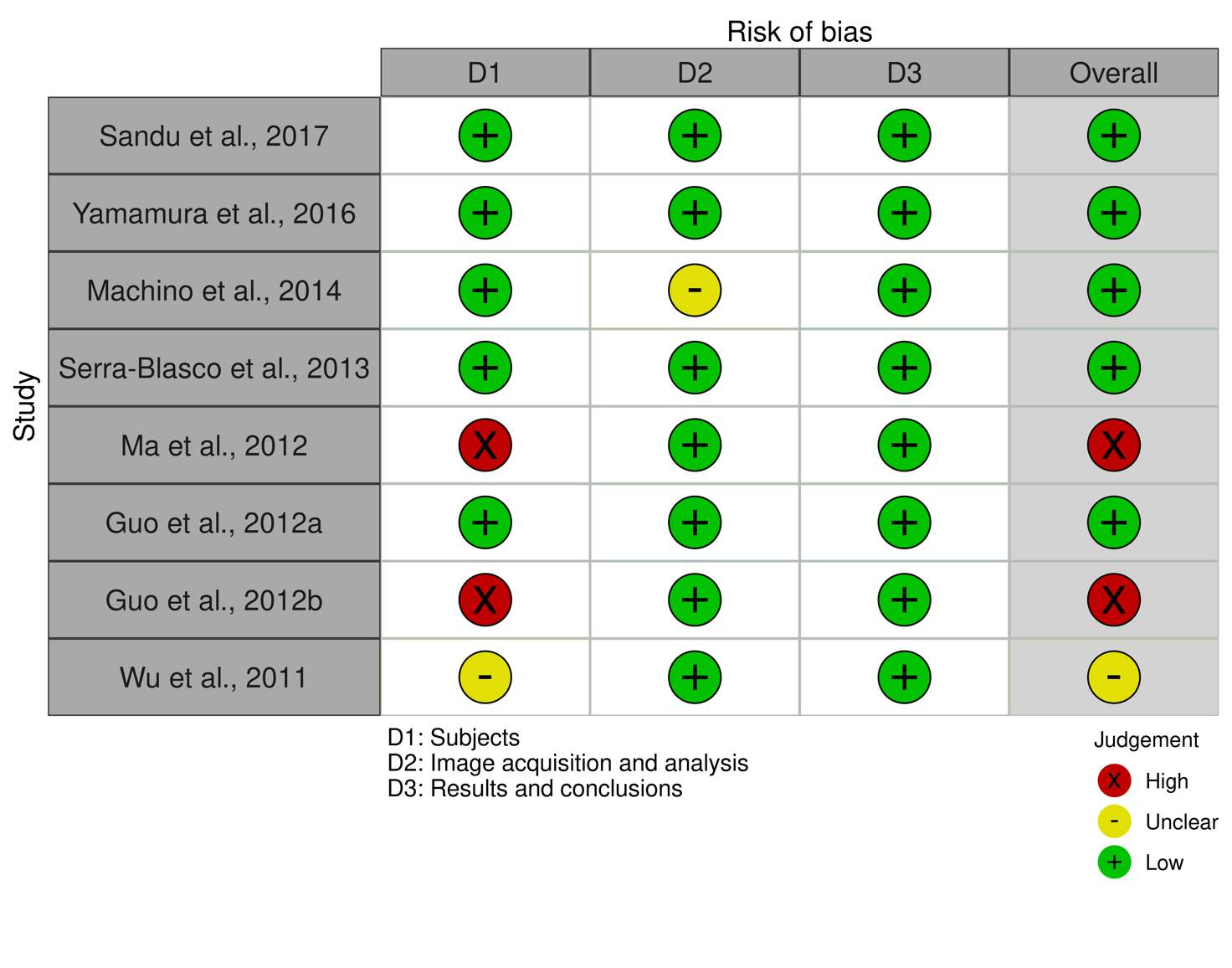


**Section 1.** Patient comorbidities in the included studies

In detail, Sandu et al. (2017) reported as exclusion criteria the age >65 years, pregnancy, previous alcohol or substance abuse in the past six months, electroconvulsive therapy treatment in the past six months, any current medical condition including neurological disorders or substantial brain damage, along with any contraindication to magnetic fields (Sandu et al., 2017). Two studies defined any serious medical/neurological comorbidity, intellectual disability or substance [ab]use in the previous six months as reasons for exclusion from recruitment (Guo et al., 2012c, 2012d). Similarly, in the study conducted by Serra-Blasco and colleagues (2013), participants with a history of head injury, neurological illness, and alcohol or substance abuse were excluded from the study (Serra-Blasco et al., 2013). Again, Wu et al. (2011) excluded participants who had any medical/psychiatric comorbidity (as well as previous head trauma with loss of consciousness), with an explicit statement that any anxiety disorder was also a reason for exclusion. Still, laxer criteria were applied for substance [mis]use or psychiatric treatment (no exposure to psychoactive medications/drugs in the previous two months) (Wu et al., 2011). Moreover, in the study by Ma and colleagues (2012), any comorbidity with bipolar disorder, a history of major illnesses, and cardiovascular disease were listed as exclusion criteria (Ma et al., 2012). Machino et al. (2014) excluded any participant with a current or previous diagnosis of psychosis, neurological disorder, severe somatic disease, intellectual disability, comorbidity with personality disorder, substance use, and a high risk of suicide (Machino et al., 2014). Yamamura et al. (2016) reported a similar list of comorbidities as exclusion criteria (Yamamura et al., 2016).

**
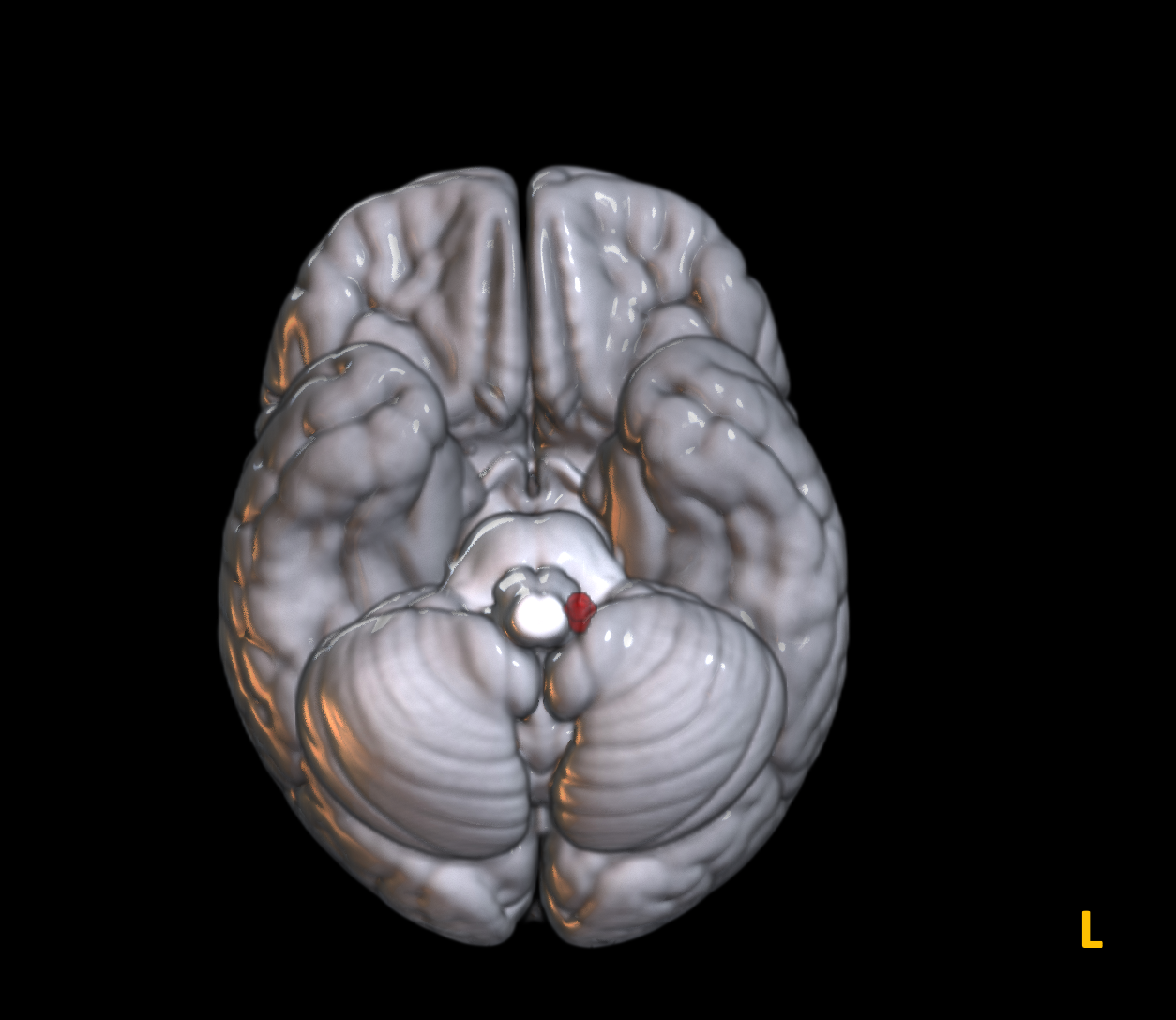
eFigure 2.** Convergent intrinsic neural activity changes in Treatment-resistant depression (TRD).

L = left. Significant convergence of intrinsic neural activity changes estimated using activation likelihood estimation algorithm on resting-state functional magnetic resonance imaging indexes, including low-frequency oscillations and regional homogeneity, in patients with TRD relative to healthy controls. A cluster in the left cerebellum/pons was found (at x,y,z [-10;-36;-32], here reported in red; p = 4*10^-6^). The probability map is rendered on a standard brain using the Montreal Neurological Institute (MNI) atlas. The rendering was created using MricroGl.

**References:**

Abdallah, C. G., Jackowski, A., Sato, J. R., Mao, X., Kang, G., Cheema, R., Coplan, J. D., Mathew, S. J., & Shungu, D. C. (2015). Prefrontal cortical GABA abnormalities are associated with reduced hippocampal volume in major depressive disorder. *European Neuropsychopharmacology: The Journal of the European College of Neuropsychopharmacology*, *25*(8), 1082–1090. https://doi.org/10.1016/j.euroneuro.2015.04.025

Carceller-Sindreu, M., de Diego-Adeliño, J., Serra-Blasco, M., Vives-Gilabert, Y., Martín-Blanco, A., Puigdemont, D., Álvarez, E., Pérez, V., & Portella, M. J. (2015). Volumetric MRI study of the habenula in first episode, recurrent and chronic major depression. *European Neuropsychopharmacology: The Journal of the European College of Neuropsychopharmacology*, *25*(11), 2015–2021. https://doi.org/10.1016/j.euroneuro.2015.08.009

de Kwaasteniet, B. P., Rive, M. M., Ruhé, H. G., Schene, A. H., Veltman, D. J., Fellinger, L., van Wingen, G. A., & Denys, D. (2015). Decreased Resting-State Connectivity between Neurocognitive Networks in Treatment Resistant Depression. *Frontiers in Psychiatry*, *6*, 28. https://doi.org/10.3389/fpsyt.2015.00028

Geugies, H., Opmeer, E. M., Marsman, J. B. C., Figueroa, C. A., van Tol, M. J., Schmaal, L., van der Wee, N. J. A., Aleman, A., Penninx, B. W. J. H., Veltman, D. J., Schoevers, R. A., & Ruhé, H. G. (2019). Decreased functional connectivity of the insula within the salience network as an indicator for prospective insufficient response to antidepressants. *NeuroImage. Clinical*, *24*, 102064. https://doi.org/10.1016/j.nicl.2019.102064

Gong, Q., Wu, Q., Scarpazza, C., Lui, S., Jia, Z., Marquand, A., Huang, X., McGuire, P., & Mechelli, A. (2011). Prognostic prediction of therapeutic response in depression using high-field MR imaging. *NeuroImage*, *55*(4), 1497–1503. https://doi.org/10.1016/j.neuroimage.2010.11.079

Grieve, S. M., Korgaonkar, M. S., Etkin, A., Harris, A., Koslow, S. H., Wisniewski, S., Schatzberg, A. F., Nemeroff, C. B., Gordon, E., & Williams, L. M. (2013). Brain imaging predictors and the international study to predict optimized treatment for depression: Study protocol for a randomized controlled trial. *Trials*, *14*, 224. https://doi.org/10.1186/1745-6215-14-224

Guo, W., Liu, F., Xue, Z., Gao, K., Liu, Z., Xiao, C., Chen, H., & Zhao, J. (2013a). Decreased interhemispheric coordination in treatment-resistant depression: A resting-state fMRI study. *PloS One*, *8*(8), e71368. https://doi.org/10.1371/journal.pone.0071368

Guo, W., Liu, F., Xue, Z., Gao, K., Liu, Z., Xiao, C., Chen, H., & Zhao, J. (2013b). Abnormal resting-state cerebellar-cerebral functional connectivity in treatment-resistant depression and treatment sensitive depression. *Progress in Neuro-Psychopharmacology & Biological Psychiatry*, *44*, 51–57. https://doi.org/10.1016/j.pnpbp.2013.01.010

Guo, W., Liu, F., Chen, J., Gao, K., Xue, Z., Xu, X., Wu, R., Tan, C., Sun, X., Liu, Z., Chen, H., & Zhao, J. (2012c). Abnormal neural activity of brain regions in treatment-resistant and treatment-sensitive major depressive disorder: A resting-state fMRI study. *Journal of Psychiatric Research, 46*(10), 1366–1373. https://doi.org/10.1016/j.jpsychires.2012.07.003

Guo, W., Liu, F., Xue, Z., Xu, X., Wu, R., Ma, C., Wooderson, S. C., Tan, C., Sun, X., Chen, J., Liu, Z., Xiao, C., Chen, H., & Zhao, J. (2012d). Alterations of the amplitude of low-frequency fluctuations in treatment-resistant and treatment-response depression: A resting-state fMRI study. *Progress in Neuro-Psychopharmacology & Biological Psychiatry, 37*(1), 153–160. https://doi.org/10.1016/j.pnpbp.2012.01.011

He, Z., Cui, Q., Zheng, J., Duan, X., Pang, Y., Gao, Q., Han, S., Long, Z., Wang, Y., Li, J., Wang, X., Zhao, J., & Chen, H. (2016). Frequency-specific alterations in functional connectivity in treatment-resistant and -sensitive major depressive disorder. *Journal of Psychiatric Research*, *82*, 30–39. https://doi.org/10.1016/j.jpsychires.2016.07.011

Hou, Z., Gong, L., Zhi, M., Yin, Y., Zhang, Y., Xie, C., & Yuan, Y. (2018). Distinctive pretreatment features of bilateral nucleus accumbens networks predict early response to antidepressants in major depressive disorder. *Brain Imaging and Behavior*, *12*(4), 1042–1052. https://doi.org/10.1007/s11682-017-9773-0

Jia, Z., Peng, W., Chen, Z., Sun, H., Zhang, H., Kuang, W., Huang, X., Lui, S., & Gong, Q. (2017). Magnetization Transfer Imaging of Treatment-resistant Depression. *Radiology*, *284*(2), 521–529. https://doi.org/10.1148/radiol.2017160820

Li, C.-T., Su, T.-P., Wang, S.-J., Tu, P.-C., & Hsieh, J.-C. (2015). Prefrontal glucose metabolism in medication-resistant major depression. *The British Journal of Psychiatry: The Journal of Mental Science*, *206*(4), 316–323. https://doi.org/10.1192/bjp.bp.113.140434

Li C.-T., Lin C.-P., Chou K.-H., Chen I.-Y., Hsieh J.-C., Wu C.-L., Lin W.-C. Su T.-P. (2010) Structural and

cognitive deficits in remitting and non-remitting recurrent depression: a voxel-based morphometric study.

*Neuroimage*. 2010; 50: 347–56. https://doi.org/10.1016/j.neuroimage.2009.11.021

Liu, F., Guo, W., Yu, D., Gao, Q., Gao, K., Xue, Z., Du, H., Zhang, J., Tan, C., Liu, Z., Zhao, J., & Chen, H. (2012). Classification of different therapeutic responses of major depressive disorder with multivariate pattern analysis method based on structural MR scans. *PloS One*, *7*(7), e40968. https://doi.org/10.1371/journal.pone.0040968

Lui, S., Wu, Q., Qiu, L., Yang, X., Kuang, W., Chan, R. C. K., Huang, X., Kemp, G. J., Mechelli, A., & Gong, Q. (2011). Resting-state functional connectivity in treatment-resistant depression. *The American Journal of Psychiatry*, *168*(6), 642–648. https://doi.org/10.1176/appi.ajp.2010.10101419

Ma, C., Ding, J., Li, J., Guo, W., Long, Z., Liu, F., Gao, Q., Zeng, L., Zhao, J., & Chen, H. (2012). Resting-state functional connectivity bias of middle temporal gyrus and caudate with altered gray matter volume in major depression. *PloS One*, *7*(9), e45263. https://doi.org/10.1371/journal.pone.0045263

Machino, A., Kunisato, Y., Matsumoto, T., Yoshimura, S., Ueda, K., Yamawaki, Y., Okada, G., Okamoto, Y., & Yamawaki, S. (2014). Possible involvement of rumination in gray matter abnormalities in persistent symptoms of major depression: An exploratory magnetic resonance imaging voxel-based morphometry study. *Journal of Affective Disorders, 168*, 229–235. https://doi.org/10.1016/j.jad.2014.06.030

Sandu, A.-L., Artiges, E., Galinowski, A., Gallarda, T., Bellivier, F., Lemaitre, H., Granger, B., Ringuenet, D., Tzavara, E. T., Martinot, J.-L., & Paillère Martinot, M.-L. (2017). Amygdala and regional volumes in treatment-resistant versus nontreatment-resistant depression patients. *Depression and Anxiety, 34*(11), 1065–1071. https://doi.org/10.1002/da.22675

Serra-Blasco, M., Portella, M. J., Gómez-Ansón, B., de Diego-Adeliño, J., Vives-Gilabert, Y., Puigdemont, D., Granell, E., Santos, A., Alvarez, E., & Pérez, V. (2013). Effects of illness duration and treatment resistance on grey matter abnormalities in major depression. *The British Journal of Psychiatry: The Journal of Mental Science, 202*, 434–440. https://doi.org/10.1192/bjp.bp.112.116228

Shah, P. J., Glabus, M. F., Goodwin, G. M., & Ebmeier, K. P. (2002). Chronic, treatment-resistant depression and right fronto-striatal atrophy. *The British Journal of Psychiatry: The Journal of Mental Science*, *180*, 434–440. https://doi.org/10.1192/bjp.180.5.434

Soriano-Mas, C., Hernández-Ribas, R., Pujol, J., Urretavizcaya, M., Deus, J., Harrison, B. J., Ortiz, H., López-Solà, M., Menchón, J. M., & Cardoner, N. (2011). Cross-sectional and longitudinal assessment of structural brain alterations in melancholic depression. *Biological Psychiatry*, *69*(4), 318–325. https://doi.org/10.1016/j.biopsych.2010.07.029

Wu, Q.-Z., Li, D.-M., Kuang, W.-H., Zhang, T.-J., Lui, S., Huang, X.-Q., Chan, R. C. K., Kemp, G. J., & Gong, Q.-Y. (2011). Abnormal regional spontaneous neural activity in treatment-refractory depression revealed by resting-state fMRI. *Human Brain Mapping, 32*(8), 1290–1299. https://doi.org/10.1002/hbm.21108

Yamamura, T., Okamoto, Y., Okada, G., Takaishi, Y., Takamura, M., Mantani, A., Kurata, A., Otagaki, Y., Yamashita, H., & Yamawaki, S. (2016). Association of thalamic hyperactivity with treatment-resistant depression and poor response in early treatment for major depression: A resting-state fMRI study using fractional amplitude of low-frequency fluctuations. *Translational Psychiatry, 6*, e754. https://doi.org/10.1038/tp.2016.18

Zhang, A., Li, G., Yang, C., Liu, P., Wang, Y., Kang, L., Wang, Y., & Zhang, K. (2019). Alterations of amplitude of low-frequency fluctuation in treatment-resistant versus non-treatment-resistant depression patients. *Neuropsychiatric Disease and Treatment*, *15*, 2119–2128. https://doi.org/10.2147/NDT.S199456
